# Predicting malnutrition‐based anemia in geriatric patients using machine learning methods

**DOI:** 10.1111/jep.14142

**Published:** 2024-09-23

**Authors:** Mehmet Göl, Cemal Aktürk, Tarık Talan, Mehmet Sait Vural, İbrahim Halil Türkbeyler

**Affiliations:** ^1^ Department of Physiology Faculty of Medicine, Gaziantep Islam Science and Technology University Gaziantep Turkey; ^2^ Department of Computer Engineering Faculty of Engineering and Natural Sciences, Gaziantep Islam Science and Technology University Gaziantep Türkiye; ^3^ Division of Geriatrics, Department of Internal Medicine Faculty of Medicine, Gaziantep Islam Science and Technology University Gaziantep Turkey

**Keywords:** anemia, artificial intelligence, J48, machine learning, Random Forest

## Abstract

**Background:**

Anemia due to malnutrition may develop as a result of iron, folate and vitamin B12 deficiencies. This situation poses a higher risk of morbidity and mortality in the geriatric population than in other age groups. Therefore, early diagnosis of anemia and early initiation of treatment is very important. This study aims to predict the diagnosis of anemia with using machine learning (ML) methods in geriatric patients followed in an outpatient clinic.

**Methods:**

In line with the purpose of the study, anemia classification was made by analysing patients' hemogram and biochemistry blood values and medical data such as malnutrition, physical and cognitive activity scores with ML methods.

**Results:**

In our data set consisting of 438 patient observations, the most successful ML algorithm was the J48 algorithm with 97.77% accuracy. In the continuation of the study, the predictive performance of anemia was investigated by excluding blood values and selecting only attributes consisting of malnutrition and physical activity scores. In this case, the most successful prediction was obtained with the Random Forest algorithm with 85.39% accuracy.

**Conclusions:**

The study showed that anemia can be predicted with high accuracy in geriatric patients without hemogram data. Additionally, our geriatric data set was shared with researchers for future research. Thus, it has contributed to the literature by opening a new path for studies on subjects such as comparing classification performances with new methodologies or predicting different diseases in geriatric patients.

## 1 INTRODUCTION

Anemia is a haematological condition that occurs due to insufficient amount of red blood cells or haemoglobin in the blood and is quite common around the world. This disease can cause serious health problems that can be life‐threatening, especially in children, pregnant and the elderly. It can significantly reduce the quality of life. Anemia in the geriatric patients focused on in this study can generally occur due to various factors such as malnutrition, chronic diseases or side effects of long‐term medications. Anemia due to malnutrition may develop as a result of iron, folate and vitamin B12 deficiencies and is frequently encountered in the elderly population. This situation leads to a decrease in physical and cognitive functions in the elderly, fatigue, decrease in bone density, falls and fractures, cardiovascular diseases and an increased risk of mortality.^1^, ^2^

The aging process causes various biological and physiological changes in the human body, and these changes have a significant impact on the health status of individuals in old age. One of the health problems frequently seen in the geriatric population is anemia.^3^ Anemia is a life‐threatening condition in elderly individuals, predisposing them to various diseases and reducing the quality of life due to these diseases, as well as increasing the risks of morbidity and mortality.^4^ Malnutrition is also seen as one of the main causes of anemia that can occur in older ages. Inadequate and unbalanced nutrition, medications used for long periods of time for chronic diseases, and the presence of some diseases can cause deficiency of nutrients such as iron, vitamin B12 and folic acid in the elderly, and therefore anemia.^5^

As with other diseases, early diagnosis and treatment of anemia is of great importance to prevent the risks that the disease may pose. At this point, traditional diagnostic methods may not always be effective enough, which may cause anemia cases to be overlooked and anemia to cause different chronic diseases in patients. In this case, machine learning (ML) techniques emerge as a powerful tool and enable the development of more precise and reliable prediction models by analysing large data sets. Because ML is an artificial intelligence technique that plays an important role in early detection and accurate diagnosis of diseases by enabling in‐depth examination of clinical data and identification of complex patterns.^6^ The use of ML in the field of medicine has brought many innovations such as improving disease diagnosis processes, early detection of life‐threatening diseases such as cancer, and personalisation of treatment plans. In particular, ML algorithms play an important role in predicting diseases and identifying risk factors associated with diseases.^7^ These algorithms allow accurate, fast and effective predictions to be made by taking input from various data sources such as patients' medical history, laboratory results and other clinical data and analysing the data.

In this study, the predictive effectiveness of various ML algorithms in diagnosing anemia is investigated using data such as blood values, malnutrition and physical activity scores of geriatric patients. In the second part of the article, current anemia classification studies in the literature will be included and the applications and potential benefits of ML techniques in this field will be discussed. The third section includes the methodology of our study, and in this section, the features of our data set and the ML algorithms used are mentioned. In the fourth section, the findings of the classification algorithms are presented. The fifth and last chapter includes the discussion and conclusion, where the results of our study and its contributions to future research are mentioned.

## 2 LITERATURE REVIEW

According to the World Health Organisation (WHO), 30% of women aged 15−49 years, more than 40% of pregnant and 40% of children aged 6−59 months worldwide are anemic.^8^ More than 115.000 maternal deaths and 591.000 prenatal deaths occur annually worldwide due to anemia. According to WHO, anemia is considered to be a haemoglobin level in the blood below 13 g/dL in men over 15 years of age, less than 12 g/dL in nonpregnant over 15 years of age, and less than 11 g/dL in pregnant.^9^ It is vitally important to detect anemia quickly and effectively to reduce anemia‐related deaths and prevent secondary diseases caused by anemia. For this reason, researchers in the literature have used artificial intelligence methods to detect anemia and classify anemia disease. When the studies are examined, they generally consider biochemistry blood test results as basic input data. For example, Geetha et al.^47^ studied the classification of red blood cells using Lasso and Ridge regression methods to detect anemia. Researchers emphasised that the Ridge regression model provides classification with better accuracy. On the other hand, Dejene et al.^10^ aimed to estimate the anemia level of pregnant in Ethiopia in their study. For this purpose, researchers have used ML methods such as Decision Tree (DT), Random Forest (RF), cat boost, and extreme gradient boosting (GB). As a result of the research, it was concluded that the best performance was the cat boost algorithm. Susič et al.^11^ estimated the possible anemia 6 weeks after birth with various ML methods to detect postpartum anemia. The ML models used by researchers are Linear regression, Kernel Ridge, Elastic Net regression, Bayesian ridge regression, Support vector regression, GB regressor, Light GB machine, extreme grading boosting regressor (XGB), and Cat Boost regressor.

Sridevi et al.^12^ conducted a comparative study on the prediction of chronic kidney disease, hypertension, diabetes and anemia. Using five different ML models, the researchers tested each data set separately before and after feature engineering. The model that best predicted anemia was the XGB method. The average age of the people in the data set used by the researchers is 51.42. Another study investigated the effectiveness of ML algorithms in the morphological classification of anemia.^13^ In addition, Pullakhandam and McRoy^14^ used ML to classify iron deficiency anemia in the US NHANES data set, which contains more than 19.000 samples. The researchers revalidated the results with the same model on an unused data set in Kenya. The ML algorithms used by researchers are K‐Nearest Neighbour (K‐NN), Naïve Bayes (NB), Multilayer Perceptron (MLP), RF, and Support Vector Machine (SVM).

In their ML studies investigating anemia in children, Khan et al.^15^ evaluated anemia status in children under 5 years of age with ML algorithms using common risk factors as features. Researchers have worked with various ML algorithms such as Linear discriminant analysis, classification and regression trees, KNN, SVM, RF and LoR. The data set used in the study was obtained from the Bangladesh Demographic and Health Survey in 2011. Farzaliyev et al.^16^ investigated ensemble learning techniques to predict anemia in children. Researchers have tested ML algorithms such as DT, SVM, RF, LoR, KNN with data sets. The data set the researchers used includes patient observations collected from Iraq's Haditha General Hospital and clinics. According to the results of the researchers, it is understood that ensemble learning techniques perform lower than individual classifiers. In another study, the prediction of anemia in children under the age of five in Afghanistan was investigated with five different ML algorithms.^17^ The ML algorithms used by researchers are KNN, NB, MLP, RF and SVM. The data set collected by the researchers with a 26‐question face‐to‐face survey includes a total of 350 records, 175 of which are anemia and 175 are nonanemia. RF was measured as the best classifier with an accuracy rate of 86.4%.

Artificial intelligence studies examining anemia associated with malnutrition are also included in the literature. Kilicarslan et al.^18^ conducted a study to predict patients with hemoglobin (HGB) anemia, nutritional anemia, and nonanemia. Researchers have estimated nutritional anemia as iron deficiency anemia, B12 deficiency anemia, and folate deficiency anemia. When they used genetic algorithm (GA) and deep learning algorithms (Stacked Autoencoder [SAE] and Convolutional Neural Network [CNN]) as a hybrid method and compared the performance of GA‐SAE and GA‐CNN models, the best prediction accuracy was obtained as 98.5% with GA‐CNN. They did. The data set they used in the research was collected from medical school patients and consists of 15,300 records consisting of 24 features and five classes. Researchers excluded pregnant, children, and cancer patients from these data. Joseph et al.^19^ conducted a study with ML algorithms to detect features related to malnutrition and associate them with anemia in the Indian Demographic and Health Survey (IDHS) data set. The researchers conducted a clustering study that reduced the data set of 1352 features to 329 features associated with anemia and malnutrition.

Pan et al.^20^ created an ML model to evaluate the risk of iron deficiency anemia 12 months after the operation in women who underwent sleeve gastrectomy in China. For this purpose, data from 407 patients aged between 26 and 36 were analysed. Qasrawi et al.^21^ investigated the relationships between students' nutritional intake and anemia with ML techniques in a sample of 755 female university students in Palestine. Researchers used K‐means clustering analysis algorithm and DT algorithm to determine the relationship between anemia and vitamin and mineral intake. According to the research results, in addition to nutrients such as folate, Vitamin B6, C, B12 or Fe, which are commonly known to be associated with anemia, choline, Vitamin E, B2, Zn, Mg were also associated with anemia. In addition, researchers emphasised that Mn and phosphorus may also be associated with the development of anemia.

Studies on the prediction of anemia have been compiled and listed in Table 1. In Table 1, the authors of the studies, the titles of the studies, the algorithm that gives the best performance in classification, the classification accuracies and the number of observations of the data set used are presented. When Table 1 is examined, it is understood that the algorithms classify anemia with accuracy rates between 68.53% and 98.5%. It is understood that the ones that offer the best performance are generally RF, DT and Boosting Algorithms. The number of observations in the data set used in the studies varies between 224 and 29.104. It is understood that the data sets consist of children, pregnant, female and adults.

**Table 1:** Anemia classification studies with ML.

Authors	Best classification algorithm	Data set records (subject)	Performance (accuracy %)
Khan et al.^15^	Random Forest	2013 Children	68.53
Farzaliyev et al.^16^	Decision Tree	600 Children	98
Zahirzada et al.^17^	Random Forest	350 Children	86.4
Kilicarslan et al.^18^	Genetic algorithm and Convolutional Neural Network	15.300	98.5
Pan et al.^20^	Support Vector Machine	407 Pregnant	79.9
Qasrawi et al.^21^	DeCision Tree	755 female	82.1
Dejene et al.^10^	Cat boost	29.104 Pregnant	97.6
Susič et al.^11^	‐	224 female	
Sridevi et al.^12^	XGboost	400	88.8
Pullakhandam and McRoy^14^	Gradient boosting	19,000	87

Abbreviation: ML, machine learning.

Various data mining techniques have been used in the literature to predict malnutrition anemia, especially iron deficiency anemia. However, not every algorithm performs well for every data set, and therefore new techniques need to be developed. Because the features of each data set are different and the size of the data set, that is, the number of records and the number of parameters, is different. The aim of the study is to predict whether geriatric patients have anemia. For this purpose, the presence of anemia diagnosis was estimated based on our original data obtained from our patients' files. In the continuation of the study, the prediction of anemia from nutritional and physical life activity scores is investigated. While this study comparatively presents classification performances with ML algorithms, it also presents an original data set containing blood values, nutritional attributes and physical activity scores of geriatric patients.

This study proposes an approach to detect anemia from nutrition and physical life activities by investigating the classification performance of various ML algorithms in the diagnosis of anemia. The contributions of the study to the literature are listed below:

- It allows a comprehensive analysis of anemia classification accuracy with ML methods. In this context, predicting the diagnosis of anemia in elderly patients with ML can improve the quality of healthcare and positively affect patients' quality of life.
- It is aimed to contribute to the field by presenting the classification performance of anemia diagnosis by selecting features related to nutrition and physical life activities.
- It also provides researchers and field experts with new ideas to explore classification problem solutions in other medical fields by sharing our data set of geriatric patients or as the potential to use new methodologies in anemia classification.
- In conclusion, the use of ML algorithms in diagnosing anemia with the nutrition and physical activity data set of geriatric patients offers an important innovation both clinically and academically.
- The findings obtained in this study may provide guidance in both medical practices and further research and may make important contributions to the health management of the elderly population.

## 3 METHOD

**Figure 1:**
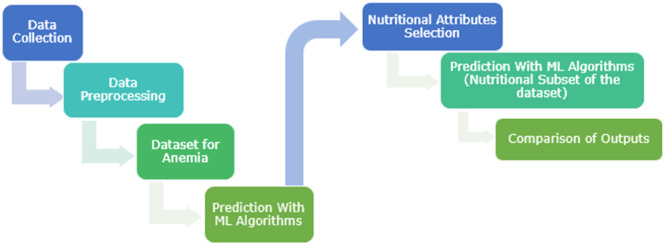
Data set creation and classification process with ML algorithms. ML, machine learning.

The flow diagram of the study's data set creation process and classification processes is shown in Figure 1. First of all, the data recorded in the patient files were collected and the preprocessing process was completed by transferring these data to digital media. At this stage, patient records with missing information were removed and the types of data (ordinal, nominal, etc.) were arranged to ensure that the classification algorithms worked more effectively. In the last case, our anemia data set emerged. Classification operations were performed with our anemia data set through ML algorithms, and the error metrics and accuracy parameters of each method were recorded. In the continuation of the study, a subset was created from the data set by selecting attributes consisting of nutrition and physical life activities. The findings were evaluated by re‐running the ML algorithms with the current data set, which did not include haemoglobin values and other blood data.

### 3.1 Creating the data set

#### 3.1.1 Data collection process

The patient data used in our study was obtained by retrospective file scanning method of comprehensive geriatric evaluation results of patients followed between 2017 and 2020 in the Geriatrics Polyclinic of a public hospital in Gaziantep, Republic of Turkey. For this purpose, Research Application Consent was received from Gaziantep Islam Science and Technology University Coordinatorship of Local Ethics Committee for Non‐Interventional Clinical Research on 13.07.2021, and the necessary ethics permission procedures were completed.

During the data collection process of the study, data from a total of 438 patients, 189 men and 249 women, were accessed and compiled. The age range of the patients in the data set is between 65 and 90, and the average age is 71.76.

#### 3.1.2 Data preprocessing and creation of the anemia data set

Our data set obtained from scanned patient data consists of 438 observations and a total of 65 attributes of these observations. One of the 65 attributes, 1 is anemia class and 37 of them are medical data related to patients' mental, physical and nutritional data. The remaining of 22 attributes consist of hemogram and biochemistry tests, and 5 of them consist of the demographic characteristics of the patients. The prominent features related to nutrition and physical activity in the Data Set are listed as malnutrition, diagnosis, number of medications used, smoking‐alcohol habit, exercise work status, calf circumference (cm), body mass index (kg/m^2^), walking speed (m/s), muscle mass (kg/m^2^), hand strength (kg), daily basic living activity score, instrumental living activity score, clock drawing, minimental test score, Yesavage depression score, standardised minimental test score, mini nutritional assessments, Tinetti balance and walking test, timed up and go test, eating. They are listed as attitude test, swallowing function test, sleep quality index. The values of categorical variables such as smoking and alcohol use recorded as “unknown, yes, no” are coded as “0, 1, 2” respectively. Similarly, values in the malnutrition category (current, risky, normal) are coded as “1, 2, 3”. The previous diagnoses of the patients (diabetes mellitus, hypertension, hyperlipidemia, cardiovascular system, thyroid, Parkinson's, rheumatological, gastrointestinal system, haematological, Alzheimer's, Asthma, Epilepsy, Depression, osteoporosis) were also coded with the corresponding numerical values.

During the data preprocessing process, the types of categorical and numerical variables were checked and incorrect preliminary definitions were corrected. Since the class variable value was unknown in 34 of the 438 data, these observations were excluded from the study. ‘Attribute Selection, Numeric to Nominal, Ordinal to Numeric, Remove Misclassified, Remove with Values’ filters were used for operations such as feature selection, type conversion, and deletion of missing data during the data preprocessing process. In the final case, ML algorithms were run with a data set consisting of 404 valid records. The data set we use in our study is shared on the Kaggle platform in csv format.^22^

### 3.2 ML methods

ML methods consist of two stages: training and testing the system based on an existing problem. In the training phase, the selected ML method performs calculations by building a model over a data set or by monitoring the behaviour of network traffic over a period of time. In the testing phase, a test data sample is given as input to the model created as a result of training, and the test data contents are classified as normal or abnormal according to the learned behaviour.^23^ With the development of artificial intelligence, its use in the medical field is increasing day by day. It is understood from the studies that the needs for object‐disease recognition and human movement perception are successfully integrated with artificial intelligence methods such as classification and clustering.^24^, ^25^, ^26^

K‐NN, NB, DT, Decision Table, SVMs, Logistic Regression, RF, CNNs and Boosting are the current ML algorithms used in anemia classification.^14^, ^27^, ^28^, ^29^, ^30^, ^31^, ^32^, ^33^ In our study; Bayes Network (BayesNet) Classifier, Decision Table, Decision Table‐NB Hybrid, Naive Bayes, RF algorithms were used.

#### 3.2.1 BayesNet Classifier

BayesNet is a classification method based on statistical modelling of data. BayesNet, one of the supervised classification algorithms, is a probability model that represents possible dependencies between variables. BayesNet calculates the probabilities to make a certain inference and then predicts the class that best fits the given input.^34^

#### 3.2.2 Decision Table

Decision Table, a classification model, is a decision‐making tool that contains all the logical conditions and results in the rows and columns of the data set as a table. Each row represents a combination of a set of conditions and the actions or decisions that will be taken if those conditions are met. Conditions and results are determined according to certain attributes, and the possible situations of each condition are shown in different columns of the table.^35^, ^36^ The Decision Table simplifies complex decision‐making processes by presenting data in a structured and easily interpretable format. This method is widely used in the field of ML and data mining for decision‐making tasks such as medical diagnosis.

#### 3.2.3 Decision Table NB Hybrid (DTNB)

DTNB is a unified classification model and is a combination of Decision Tables and NB algorithm. This method combines the classification accuracy of NB with the flexibility of Decision Tables. Decision Tables outperform NB on incomplete data sets in some cases, while NB is known for its ability to model the interdependence of certain features. It uses a forward selection search that aims to select the best features at each step of DTNB. In this search process, NB evaluates the impact of each feature independently on classification, while Decision Table analyzes the performance of combinations of features.^37^, ^38^

#### 3.2.4 J48

Based on C4.5 algorithms, J48 is a DT algorithm used in data mining. J48 aims to create a tree by calculating Entropy and Gain values for the data set. The algorithm calculates the information gain for all attributes and creates the node of the tree by selecting the attribute with the highest gain. This process continues sequentially until the leaf nodes of the tree are reached by selecting the best feature at each node and dividing the data.^36^, ^39^

#### 3.2.5 NB

Based on Bayes theorem, NB is a probabilistic classification algorithm. This algorithm is widely used, especially in natural language processing tasks such as text classification and spam filtering.^40^ NB uses probability calculations to determine the decision with the highest probability.^41^ This method assumes that each feature is independent of the others. The classifier determines the probability of an event occurring by making simple probability calculations.^42^ NB is both computationally efficient and easy to implement, and can produce effective results in practice. These features make NB a preferred method in a wide range of applications.

#### 3.2.6 RF

RF is a powerful ensemble learning method that combines many DTs to improve prediction accuracy and reduce overfitting. Each DT is trained with randomly selected data subsets and features and performs classification independently. The final classification is determined by selecting the most popular class among the trees' votes.^43^ This method has gained popularity due to its high accuracy in often complex and large data sets, its effectiveness in eliminating overfitting problems commonly encountered in DTs, and its processing speed.^44^ The success of the algorithm is due to its capacity to independently construct DTs and then combine them to create a robust learner, suitable for various classification and regression tasks.^45^ Its ability to create independent DTs and combine them into a powerful learner, combined with its ability to reduce overfitting, positions it as a preferred choice for a wide range of ML tasks.

### 3.3 Measuring the classification performance

The data in the data set used in the study is expressed in two different classes, positive and negative, according to the score of the comments made. In this study, the following criteria were taken into account to evaluate the performance of ML models:

*Precision:* It expresses the ratio of true positive predictions to total positive predictions and shows how accurate positive predictions the model makes.

*Recall:* It shows how many true positive examples were predicted correctly and indicates how well the model captured all positive examples.

*F‐Measure:* It is the harmonic mean that measures the balance between precision and sensitivity and evaluates the overall performance of the model.

*ROC Area:* It represents the area under the receiver operating characteristic curve and measures the classification performance of the model, specifically the relationship between the true positive rate and false positive rate.

*PRC Area:* It refers to the area under the Precision‐Recall curve and is used to evaluate model performance, especially on unbalanced data sets.

*Accuracy:* It shows the ratio of correct predictions to total predictions and evaluates the overall success of the model.

These metrics allowed us to comprehensively evaluate how well the models performed in different aspects. The results revealed the strengths and weaknesses of each model. In the study, the effectiveness of the models was examined using these performance evaluation concepts. 10‐fold cross‐validation method was used to train the data set to increase the accuracy performance of our model. This method ensures that the model performs consistently across different data subsets and increases the generalisability of the results. Cross‐validation also shows that our model is applicable to other populations and clinical settings.

## 4 RESULTS

### 4.1 Classification findings with anemia data set

Table 2 compares the prediction accuracy performances of ML algorithms. It is clear from Table 2 that ML algorithms generally show successful prediction accuracy. When the performance of ML algorithms was compared according to accuracy parameters, the highest prediction accuracy was obtained from the J48 algorithm with 97.77%. Other performance metrics also show that J48 predicts with the highest accuracy. The DT algorithm performed almost as well as J48. DTNB, RF, and BayesNet algorithms predicted anemia with over 93% accuracy. Although the NB algorithm showed relatively lower accuracy performance than others, it still showed a successful performance with 91.3%. The average prediction accuracy of the algorithms was calculated as 95.17%. Although NB, BayesNet and RF were below this average, it can be said that the methods used in the study showed a competitive performance with an accuracy of 91% or more in detecting anemia.

**Table 2:** Accuracy performances of ML algorithms in anemia prediction.

ML algorithm	Precision	Recall	F‐measure	ROC area	PRC area	Accuracy %
J48	**0.980**	**0.978**	**0.978**	**0.980**	**0.965**	**97.7723**
DTNB	0.962	0.958	0.959	0.963	0.977	95.7921
RF	0.949	0.950	0.949	0.964	0.984	95.0495
BayesNet	0.940	0.936	0.937	0.814	0.890	93.5644
Naïve Bayes	0.914	0.913	0.914	0.849	0.871	91.3366
Decision Table	0.977	0.975	0.976	0.980	0.974	97.5248
AVG	0.953	0.951	0.952	0.925	0.943	95.1732

*Note*: Bold indicates the highest value of the best algorithm.

Abbreviations: AVG, average; DTNB, Decision Table Naïve Bayes Hybrid; ML, machine learning; PRC, Precision‐Recall Curve; ROC, receiver operating characteristic.

### 4.2 Classification findings made with malnutrition and physical activity attributes

In the second stage of the study, a new subset of the data set was created by selecting attributes related to malnutrition and physical activity tests in our data set. Prediction of anemia disease was carried out in this data set. The correlation between each attribute value in the new data set and the anemia class was evaluated with Pearson's correlation method. A correlation approach is used to determine the strength and direction of any potential connection between two variables or data set variables.

**Figure 2:**
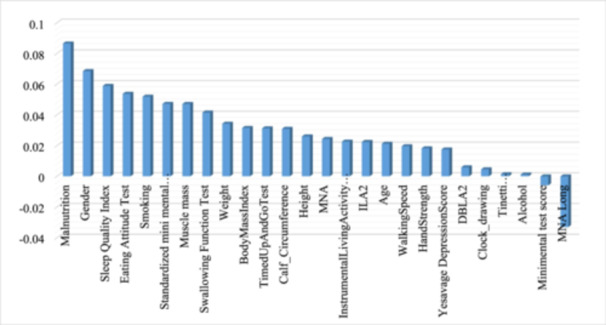
Correlation between anemia and other attributes.

The results of the correlation analysis of malnutrition and physical activity attributes with anemia class are shown in Figure 2. Figure 2 shows that malnutrition, gender, sleep quality index, eating attitude test and smoking are the strongest variables associated with anemia. In addition, it was obtained through the analysis of the data set that many attributes related to malnutrition, such as the person's weight, height, body weight index, muscle mass, smoking and alcohol use, body mass index, calf circumference, swallowing function test, are associated with anemia. Thus, the most important features of the data set are determined using correlation analysis. In the last case, the relatively important attributes of malnutrition and physical activity in the data set were identified by the diagnosis of anemia.

When the prediction accuracies of ML algorithms are examined in Table 3, the highest prediction accuracy was obtained from the RF algorithm with 85.396%. The average accuracy rate of the algorithms was approximately 84%. The algorithm that classified relatively with the least accuracy was NB with 81.68%. Although DTNB and NB offer below‐average accuracy, ML algorithms used to predict anemia from nutritional attributes have generally performed a successful classification.

**Table 3:** Accuracy performances of ML algorithms in anemia prediction with malnutrition and physical activity attributes.

ML Algorithm	Precision	Recall	F‐Measure	ROC Area	PRC Area	Accuracy %
J48	0.752	0.842	0.780	0.487	0.675	84.1584
DTNB	0.746	0.829	0.778	0.508	0.682	82.9208
RF	0.875	0.854	0.791	0.606	0.747	**85.396**
BayesNet	0.794	0.847	0.795	0.535	0.693	84.6535
Naive Bayes	0.814	0.817	0.816	0.617	0.739	81.6832
Decision Table	0.813	0.851	0.802	0.474	0.671	85.1485
AVG	0.799	0.84	0.793	0.537	0.701	83.9934

Abbreviations: AVG, average; BayesNet, Bayes Network; DTNB, Decision Table NB Hybrid; ML, machine learning; PRC, Precision‐Recall Curve; ROC, receiver operating characteristic.

## 5 DISCUSSION AND CONCLUSION

This study aims to detect malnutrition‐related anemia in geriatric patients with artificial intelligence methods and to evaluate the performance of different classification algorithms used for this purpose. In this regard, first of all, the prediction of those with anemia and those without anemia was made from the data set of our patients. In the continuation of the study, it was investigated whether anemia could be diagnosed without using blood values such as HGB and hematocrit (HCT). For this purpose, a subset of our data set consisting of nutritional attributes and physical life activity scores was selected and the same classification algorithms were run again and the findings were shared.

The results obtained as a result of the classification experiments performed on the data set reveal the performance differences and strengths/weaknesses between the algorithms. J48 and RF algorithms attract attention with the highest correct classification rate and lowest error metrics in experimental studies. These algorithms can make a simple and effective classification with high accuracy according to the characteristics of the data set. On the other hand, it can be said that all ML algorithms studied were successful in classifying anemia in our data set. Differences between the success performance of the algorithms may be related to the extent to which the attributes in the data set adapt to the data type (ordinal, nominal) and the complexity of the data set. Because experiments conducted on both data sets show that different classification algorithms exhibit different performances under different conditions.

In the correlation analysis made between the attributes in the subset selected from the data set and the anemia class, the highest correlation was found between the malnutrition variable. This confirms the relationship between anemia and malnutrition in the literature.^46^ It has also been observed that the physical characteristics of our patients, such as smoking and alcohol use, height, weight and body mass index, muscle mass, calf circumference, and parameters that reveal the loss of strength and movement due to aging, are associated with anemia. Eating attitude test, swallowing function test, timed up and go test, instrumental living activity score, walking speed and hand strength are the prominent ones. While the findings obtained from the correlation analysis reveal the relationship between anemia and nutrition and physical life activities, they also show that the age‐related movement functions of geriatric patients are indirectly related to anemia. This makes it important to regularly monitor elderly patients not only for their blood values but also for their nutritional status and physical activity capacity.

If the symptoms of malnutrition anemia are not diagnosed and treated early, shortness of breath, dizziness, lack of concentration, pale skin colour and can be seen as common laboratory findings of life‐threatening diseases.^18^ Many malnutrition studies show that approximately a quarter of the world's population is anemic.^47^ When the literature is examined, it is understood from the above‐mentioned studies that anemia studies generally focus on children, pregnant and adults. In fact, Pullakhandam and McRoy^14^ excluded patient data over the age of 60 from their study. Predicting malnutrition anemia, especially in geriatric patients, which we focused on in our study, will probably make it easier to prevent many diseases and life‐threatening situations that may arise. For this reason, the contribution of the study to the literature is very important.

This study provides a guide to make a fast and effective classification prediction using ML methods in detecting anemia specifically, and to compare the performance values of the algorithms on a specific data set. The classification accuracies obtained in the study are compatible with the results of the studies examined on malnutrition anemia.^18^, ^19^, ^20^, ^21^ In fact, the validation performance of the experimental study conducted only with nutritional attributes, without blood values such as HGB and HCT, which are direct indicators of anemia, has a higher performance than the results of other studies (Joseph et al.^19^; Pan et al.^20^; Qasrawi et al.^21^). This shows the contribution of the study to the field as it indicates that anemia can be detected without using blood values.

In our study, the use of ML algorithms in the diagnosis of anemia of malnutrition and physical activity test data of geriatric patients integrated with hemogram and biochemistry blood test results offers an important innovation both clinically and academically. The findings obtained from this study and the data presented pave the way for new studies that will guide medical practices for geriatric patients. In addition, potential studies to improve the health services and quality of life of the elderly population make significant contributions to the health management of geriatric patients. In this study, the entire data set is shared so that it can be used in future studies.^22^ With our data set, it is possible to perform prediction studies on other vital functions and nutritional status of geriatric patients in addition to anemia. For example, malnutrition and physical activity tests can be analysed with hemogram and biochemistry results using artificial intelligence techniques. In addition to using up‐to‐date ML algorithms to increase the accuracy and reliability of our model, 10‐fold cross‐validation method was also used. This method allows us to better evaluate the model performance and ensures the reliability of our findings. In future studies, the predictive performance of the model in different populations can be investigated by collecting data from different geographic regions and demographic groups. Our predictive model can use patient history, laboratory results, and treatment information from electronic health records to detect anemia. Continuous health data collection from wearable devices (e.g., smartwatches and fitness trackers) can be integrated into our model. Thus, anemia risk can be estimated instantly from data from different sources using data integration techniques. In addition, it allows researchers to increase the performance of the algorithms they use in solving classification problems, test new methods and research new ideas, especially in predicting anemia and malnutrition. In this way, a data set that can be used for both clinical data and testing purposes in artificial intelligence studies is contributed to the literature in subsequent studies.

## AUTHOR CONTRIBUTIONS

All of the authors contributed equally to this work and reviewed the manuscript.

## CONFLICT OF INTEREST STATEMENT

The authors declare no conflict of interest.

## ETHICS STATEMENT

This study was approved by Gaziantep Islam Science and Technology University Coordinatorship of Local Ethics Committee for Non‐Interventional Clinical Research on 13.07.2021, and the necessary ethics permission procedures were completed.

## Data Availability

The data that support the findings of this study are available on request from the corresponding author. The data are not publicly available due to privacy or ethical restrictions.
